# Physicochemical Characterization and Kinetics Study of Polymer Carriers with Vitamin C for Controlled Release Applications

**DOI:** 10.3390/ma17225502

**Published:** 2024-11-12

**Authors:** Magdalena Bańkosz

**Affiliations:** Cracow University of Technology, CUT Doctoral School, Faculty of Materials Engineering and Physics, Department of Material Engineering, 31-864 Cracow, Poland; magdalena.bankosz@pk.edu.pl

**Keywords:** kinetic modeling, vitamin C, controlled release, microcapsules, hydrogels

## Abstract

This study focuses on the selection and evaluation of a kinetic model for the release of vitamin C from different delivery systems, including microcapsules, hydrogels, and a hybrid system combining both. The microcapsules were synthesized from a 2% sodium alginate solution and with vitamin C incorporated in selected formulations. Hydrogels were obtained through photopolymerization using poly(ethylene glycol) diacrylate and polyvinyl alcohol, with and without the addition of vitamin C. The hybrid system incorporated the vitamin C-containing microcapsules within the hydrogel matrix. Physicochemical properties, such as density, porosity, and water vapor transmission rate (WVTR), were evaluated. Kinetic studies of vitamin C release were conducted under dynamic and static conditions, and the experimental data were fitted to six different kinetic models: zero-order, first-order, second-order, Higuchi, Korsmeyer–Peppas, and Hixson–Crowell. The Higuchi and Korsmeyer–Peppas models provided the best fit for most systems, indicating that the release is predominantly controlled by diffusion and, in dynamic conditions, swelling of the matrix. The hybrid system, while exhibiting slower release than the microcapsules and hydrogel alone, demonstrated more controlled and sustained release, which is advantageous for applications requiring prolonged action.

## 1. Introduction

Modern pharmacokinetics and pharmaceutical technology aim to optimize the release of active substances from medicinal formulations for effective and controlled drug delivery. A well-designed delivery system impacts pharmacodynamics, bioavailability, and therapeutic efficacy. Kinetic models are crucial in studying substance release, describing mechanisms and predicting behavior under physiological conditions [1,2]. Accurate kinetic modeling enhances our understanding of drug behavior, especially in modern controlled release systems like micro- and nanocarriers [3]. The first-order model, for instance, links the release rate to the amount of substance left in the carrier, often used in polymer matrix systems with simple release profiles [4,5]. Further developments of the first-order model include more complex nonlinear models, such as the Weibull model, which is used in cases where the release is more complex and may be controlled by various mechanisms, including dissolution, diffusion, and degradation [6,7]. The Higuchi model, a classic developed in the 1960s, focuses on diffusion-limited release from porous matrices and is still widely used, particularly in semi-solid pharmaceutical forms, like creams, ointments, and transdermal patches [8,9]. Lin et al. proposed a model incorporating dissolution and diffusion from a spherical matrix, comparing it to Higuchi’s model in diffusion-controlled processes [10]. Merchant et al. explored extended-release tablets of cefpodoxime proxetil using the Higuchi model for 24 h release analysis [11]. Recently, the Korsmeyer–Peppas model has gained popularity for systems where release involves both diffusion and degradation. Its empirical equation suits both simple and complex release systems, making it widely used in nanoparticle and microparticle research [12,13,14]. Studies on the release of aspirin from PLA microparticles were also conducted, using three selected kinetic models: zero-order, first-order, and the Higuchi mode [15].

Vitamin C, which is widely used as an ingredient in dietary supplements, cosmetics, and pharmaceutical formulations, requires careful consideration in terms of release kinetics due to its sensitivity to storage and processing conditions, such as temperature, light, or oxygen [16,17,18]. Vitamin C is prone to degradation, making its stability in pharmaceutical formulations difficult to maintain. Studies on the release of vitamin C from polymeric nanoparticles have shown that the stability of the vitamin significantly increases when enclosed in controlled release systems, suggesting the need for a thorough analysis of the release kinetics of this substance [19,20,21]. Additionally, kinetic models can play a crucial role in predicting optimal storage conditions and applications of vitamin C in various products [22]. Literature data indicate various efforts to develop effective carriers for vitamin C, a compound sensitive to environmental factors, such as light, oxygen, and temperature. As a potent antioxidant, vitamin C has wide applications in pharmaceutical, food, and cosmetic products; however, its chemical instability limits both its effectiveness and bioavailability. Research efforts focus on creating carrier systems that can protect vitamin C and enable its sustained release. Previous studies illustrate a broad range of approaches in this field, though certain limitations persist. Baek et al. concentrated on developing vitamin C-containing nanocapsules using modified cellulose nanocrystals (CNC) and chitosan to enhance the longevity of vitamin C’s activity by stabilizing it within nanocapsules. This stabilization was achieved by employing phosphorylated CNC as a structural element and glycidyltrimethylammonium chloride (GTMAC) to modify chitosan. The results showed high encapsulation efficiency (90.3%) and a controlled release profile of vitamin C. However, the technology remains highly complex and costly due to the need for chitosan modification and environmental protection measures (e.g., nitrogen atmosphere), which may hinder its scalability for industrial production. The associated costs and technological requirements could present additional challenges for large-scale manufacturing [21]. In another study, Perchyonok et al. incorporated vitamin C into chitosan hydrogels as an antioxidant component. However, the study’s focus was on analyzing the release of nonsteroidal anti-inflammatory drugs (aspirin, ibuprofen, naproxen) rather than the specific release kinetics of vitamin C. This research did not address how vitamin C impacts hydrogel properties, limiting the interpretation of this approach’s effectiveness. The lack of detailed data on vitamin C release kinetics constrains the evaluation of its therapeutic potential [23]. Additionally, research by Abdullah et al. analyzed avocado seed protein-based hydrogels as vitamin C carriers, produced using various drying techniques. These hydrogels were evaluated for water retention, swelling properties, and their capacity to release vitamin C. However, under elevated temperatures (50–75 °C), an undesirable burst release of vitamin C was observed, leading to rapid depletion of the active compound and significantly reducing the carrier’s effectiveness. The variability in protein leaching, dependent on environmental parameters, suggests structural instability, posing limitations for therapeutic applications that require controlled, sustained release [24]. Hydrogel-based vitamin C carriers continue to be a subject of intensive research, as evidenced by recent publications from 2024. For instance, Szatkowski et al. investigated hydrogels as vitamin C-enriched wound dressings for treating chronic wounds. As reported, vitamin C-enriched hydrogels exhibit high thermal stability and mechanical strength, making them promising materials for clinical use. Multilayered dressings were developed, theoretically supporting the gradual release of vitamin C. However, the study did not include an analysis of vitamin C release kinetics, which significantly hampers a complete assessment of this approach’s efficacy. The lack of quantitative release data and the undefined impact of the multilayer structure on vitamin C stability underscore the need for further research to fully evaluate this solution for controlled vitamin C delivery [25].

In summary, studies on vitamin C carriers are essential and require further analysis, as existing solutions, despite their diversity, face many limitations related to the stability and control of active compound release. Current systems, such as cellulose-based nanocapsules and multilayer hydrogels, offer certain benefits but are challenged by high production costs, complex technological processes, and difficulties in achieving controlled vitamin C distribution over extended periods. The lack of precise studies on release kinetics, stability in varying environmental conditions, and long-term bioavailability of vitamin C highlights the significance of the present study. As part of this study, the synthesis of potential carriers, such as microcapsules and hydrogels containing vitamin C, was initially carried out. These systems were then combined to create hydrogels with embedded microcapsules, aiming to optimize the release kinetics and stability of the active substance. After synthesis, the materials were subjected to detailed physicochemical characterization, analyzing properties such as the swelling degree, morphology, and chemical stability. The next step involved studying the release kinetics of vitamin C and fitting the results to six different kinetic models, including first-order, Higuchi, and Korsmeyer–Peppas models. The innovative aspect of this study is the use of complex carrier systems combining microcapsules with hydrogels, which represents a novel approach to controlled vitamin C release. Most previous studies have focused on systems, such as standalone hydrogels or microcapsules, which were used for the protection and gradual release of active substances. The combination of these two types of carriers can offer significant benefits. Microcapsules act as internal reservoirs, providing better protection of vitamin C against environmental degradation, while the hydrogel, as an external matrix, can control the swelling rate and further modulate the release rate depending on environmental parameters (e.g., pH, temperature). This approach is expected to enable control of the release process at both the microcapsule and hydrogel levels. Moreover, literature data indicate a significant need for further research on the synthesis and analysis of this type of carrier, justified by the necessity to optimize their properties and effectiveness. Most available studies focus on aspects related to the degradation kinetics of vitamin C, resulting in a limited understanding of the controlled release process of this active substance. This issue requires further development, underscoring the importance of the presented study.

## 2. Materials and Methods

### 2.1. Materials

Polyvinyl alcohol (PVA, powder, Mw 13,000–23,000, 87–89% hydrolyzed), diacrylate poly(ethylene glycol) (crosslinking agent, PEGDA, average molecular weight Mn = 700 g/mol) and 2-hydroxy-2-methylpropiophenone (photoinitiator, 97%, d = 1.077 g/mL), Alginic acid sodium salt, iron(III) chloride, 1,10-fenantrolina, vitamin C (L-Ascorbic acid) were purchased from Sigma Aldrich (Saint Louis, MO, USA).

### 2.2. Synthesis of Microcapsules

To prepare the microcapsules based on sodium alginate, a 2% solution of sodium alginate was first created by dissolving the appropriate amount of alginate in distilled water, stirring until a homogeneous viscous solution was achieved. This sodium alginate solution was then slowly dripped using a syringe with a fine needle into a saturated solution of calcium chloride (CaCl_2_) to induce gelation. The formation of alginate microcapsules occurred immediately upon contact with the calcium ions, as the Na^+^ ions were replaced by Ca^2+^ ions, leading to crosslinking of the alginate.

**Figure 1:**
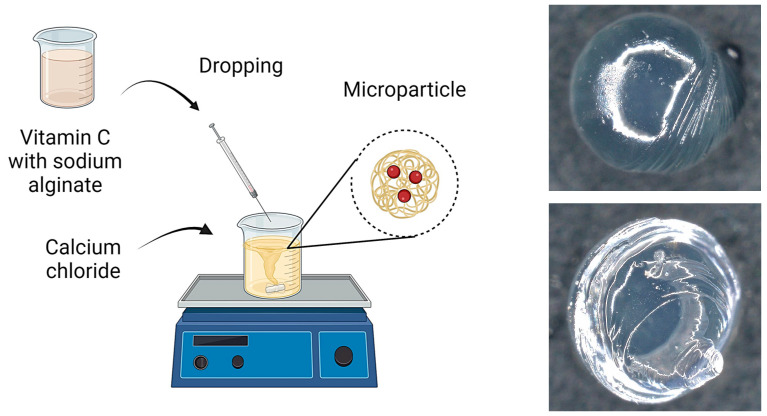
Scheme of obtaining microcapsules (**left**), sample photos (**right**).

In the second series of experiments, the sodium alginate solution was enriched by incorporating vitamin C at a concentration of 50 mg/mL. Vitamin C was dissolved directly into the 2% alginate solution, and the encapsulation process was carried out in the same manner as before by dripping into the saturated calcium chloride solution. This resulted in the formation of microcapsules containing vitamin C within the alginate matrix. After the gelation process was complete, the microcapsules from both series were centrifuged to separate them from the excess calcium chloride solution. Centrifugation was conducted at a speed of 3000 rpm for 10 min. The microcapsules were then washed with distilled water to remove any remaining Ca^2+^ ions and impurities, followed by drying using filter paper. The resulting microcapsules were subjected to further physicochemical characterization. A scheme for obtaining and a photo of sample microcapsules are presented in Figure 1.

### 2.3. Synthesis of Hydrogels

To obtain hydrogels, a photopolymerization process was used, where polyethylene glycol diacrylate served as the crosslinking agent, and 2-hydroxy-2-methylpropiophenone was used as the photoinitiator. The base polymer forming the hydrogel matrix was polyvinyl alcohol (PVA). Polyvinyl alcohol solution with a concentration of 15% was prepared by dissolving PVA powder in distilled water. First, the required amount of PVA was carefully weighed on a RadWag analytical balance (RADWAG Electronic Balances, Radom, Poland) and added to the distilled water in a beaker to obtain a final concentration of 15%. The solution was then heated to 80 °C with continuous stirring using a magnetic stirrer (IKA, Warsaw, Poland) with a hot plate. The stirring speed was set at 500 rpm to ensure even mixing and prevent PVA from clumping. The solution was maintained at this temperature and stirring speed for 45 min until the PVA was completely dissolved and a clear, homogeneous solution was obtained. After dissolution, the solution was allowed to cool to room temperature before further use in hydrogel synthesis. Next, PEGDA and the photoinitiator were added to the solution in appropriate concentrations. The mixture was stirred until homogeneous and then exposed to UV radiation to initiate the photopolymerization reaction, creating the crosslinked hydrogel network. This resulted in the formation of a basic hydrogel that served as the foundational material. In the next series of experiments, vitamin C was added to the PVA solution at a concentration of 50 mg/mL before starting the photopolymerization. The process was carried out similarly to the preparation of the hydrogel without vitamin C, leading to the formation of a hydrogel containing dissolved vitamin C. Finally, in the third series, a hybrid hydrogel was created by incorporating the previously obtained microcapsules containing vitamin C into the PVA solution. After adding the microcapsules, the entire mixture was subjected to the photopolymerization process using PEGDA and the photoinitiator, resulting in the formation of a hybrid hydrogel with embedded microcapsules containing vitamin C. In each case, the resulting hydrogels were further subjected to physicochemical characterization. A list of the samples with their identification is presented in Table 1.

### 2.4. Physicochemical Characterization

In the first stage of this study on the physicochemical properties of the hydrogels, the focus was on determining their density and porosity. For this purpose, hydrogel samples were immersed in a specific volume of isopropanol to assess how much fluid the material could absorb. After 5 min, the change in the volume of absorbed alcohol was measured. The samples were then removed from the isopropanol, and the difference in the volume of alcohol before and after removing the samples was used to calculate the density and porosity using the following equations:

$$d=\frac{m}{V_{2}-V_{3}}$$

$$P=\frac{V_{1}-V_{3}}{V_{2}-V_{3}}$$

where:

*m*—mass of the sample, g;
*V*_1_—initial volume of isopropanol, cm^3^;
*V*_2_—volume of isopropanol with the immersed sample, cm^3^;
*V*_3_—volume of isopropanol after removing the sample, cm^3^.

The next stage of this study involved analyzing the sorptive capacity of the hydrogels. After preparation and drying, the samples were accurately weighed using an analytical balance and then placed in simulated body fluid (SBF) and distilled water. The samples were incubated for varying periods: 1 h, 6 h, 12 h, and 24 h. After each incubation period, the excess fluid was removed, and the samples were reweighed to determine their mass after absorbing the fluid. Based on these measurements, the swelling coefficient was calculated using the following equation:

$$\alpha =\frac{m_{t}-m_{0}}{m_{0}}$$

where:

*α*—degree of swelling, g/g;
*m_t_*—mass of the swollen hydrogel after time t, g;
*m*_0_—initial mass of the hydrogel, g.

Each sample underwent three repetitions, and the obtained values of the swelling coefficient were averaged to provide representative data on the sorptive capacity of the hydrogel matrices. This allowed for a comparison of the absorptive properties depending on the type of incubation medium and the duration of contact with the solutions.

Next, to conduct the water vapor transmission rate (WVTR) test for hydrogels and evaluate their ability to allow water vapor permeability, which is crucial in medical applications, hydrogel samples were prepared in the form of discs with known mass and thickness. These samples were placed as covers on glass containers filled with water of a specified mass. The prepared containers with the samples were then placed in an incubator for 24 h at 37 °C. After this period, the containers were removed, and the remaining water in each container was weighed again. Based on the difference in the water mass before and after incubation, the water vapor transmission rate (WVTR) was calculated using the following formula:

$$WVTR=\frac{m_{0}-m_{t}}{A\times t}$$

where:

*WVTR*—water vapor transmission rate, g/m^2^h;
*M*_0_—initial mass of water, g;
*m_t_*—mass of water after time t, g;
*t*—time, h;
*A*—evaporation area through the sample, m^2^.

This test allowed for a precise evaluation of the vapor permeability properties of the hydrogels, which is essential for their effectiveness in applications such as medical dressings, where controlled moisture exchange is required.

As part of the physicochemical analysis, the obtained materials were evaluated using Fourier-transform infrared spectroscopy (FT-IR). The analysis was conducted using a Nicolet iS5 spectrometer (Thermo Scientific, Waltham, MA, USA). Spectra were recorded in a range of 4000–500 cm^−1^, with a resolution of 4.0 cm^−1^, under room temperature conditions. The FT-IR study allowed for the identification of functional groups present in the samples and the assessment of chemical interactions within the obtained materials.

### 2.5. Incubation Analysis in Simulated Body Fluids

In the next stage of this study, incubation was conducted for both hydrogels and microcapsules in simulated body fluid (SBF) to replicate the human body environment and evaluate the properties of both materials under these conditions. The aim of this experiment was to analyze the impact of SBF incubation on changes in pH and ionic conductivity (ELMETRON, Zabrze, Poland), which could indicate ion exchange processes, material degradation, or the release of active substances from the microcapsules. This study was carried out over 48 h, with measurements of pH and ionic conductivity taken at specific time points (0, 1, 4, 8, 12, 24, and 48 h). Calibrated pH meters and conductivity meters were used to ensure high precision of the results. Both the hydrogel and microcapsule samples were placed in glass containers filled with 50 mL SBF and incubated at 37 °C to simulate physiological conditions. Changes in pH were monitored to assess the release of ions or other substances from the hydrogel and microcapsule matrices, which could affect the stability of the surrounding environment’s pH. Simultaneously, changes in ionic conductivity allowed for the evaluation of material degradation and the ion exchange process between the microcapsules or hydrogels and the SBF. By performing regular measurements, a complete profile of these parameters over time was obtained, providing a thorough assessment of the interactions between both systems and physiological fluids, as well as their potential applications in biological environments.

### 2.6. Microscopic Observations and Surface Roughness

For further characterization of the microcapsules and hydrogels, a Keyence digital microscope was used, allowing for precise observation of surface structure and measurements of sample dimensions. The microcapsules were observed under the microscope, and their diameters were accurately measured, enabling the assessment of size distribution and uniformity of the capsules. For the hydrogels, surface roughness measurements were conducted, determining key parameters such as *R_a_* (average roughness) and *R_z_* (maximum profile height). *R_a_*, the average roughness, was evaluated as follows:

$$R_{a}=\frac{1}{L}\int_{0}^{L}\left|y\left(x\right)\right|dx$$

where:

*L* is the length of the measurement;
*y*(*x*) is the deviation of the surface profile from the mean line.

*R_z_*, the maximum profile height, was evaluated as follows:

$$R_{z}=\frac{1}{n}\overset{n}{\underset{i=1}{\sum }}\left(Z_{p}^{i}+Z_{v}^{i}\right)$$

where:

$Z_{p}^{i}$ represents the height of the highest peak;
$Z_{v}^{i}$ represents the depth of the lowest valley;
*n* is the number of measured segments.

These parameters allowed for the evaluation of the hydrogel surface topography, which is crucial for understanding their functional properties, such as their ability to interact with bodily fluids or their structural stability in a biological environment. The results obtained from digital microscopy formed a significant part of the comprehensive physicochemical analysis of the studied materials. In addition, surface morphology was carried out using scanning electron microscopy (JEOL IT200 (JEOL Ltd., Peabody, MA, USA).

### 2.7. Evaluation of Release Kinetics Under Static and Dynamic Conditions

In this study, the release of vitamin C was investigated in three types of samples: microcapsules containing vitamin C, hydrogels with vitamin C, and hybrid hydrogels that incorporated microcapsules with encapsulated vitamin C. The release of the active substance was analyzed using a spectrophotometric method based on a colorimetric reaction between the released vitamin C, ferric chloride (FeCl_3_), and 1,10-phenanthroline, forming a colored complex. This allowed for the accurate quantification of vitamin C release. The release experiments were carried out under two different conditions:

- Static release: The samples were incubated at 37 °C in a controlled incubator without agitation.
- Dynamic release: The samples were placed on a shaker at 37 °C to simulate dynamic conditions, enhancing the interaction between the samples and the fluids.

For both conditions, measurements of vitamin C release were taken at several time points: 0.5, 1, 2, 4, 6, 12, 18, 24, 36, and 48 h. In both cases, this study was conducted in simulated body fluid, with each measurement performed in triplicate. A calibration curve was constructed to quantify the concentration of vitamin C based on absorbance readings. Fluid samples were collected at each time point, and the concentration of vitamin C was analyzed to establish the release profile over time. Following the release experiments, the obtained results were fitted to six different kinetic models to identify the mechanisms controlling the release process for each type of sample. This modeling approach allowed for the evaluation of the best-fit model and the determination of the release mechanism for each system (microcapsules, hydrogels, and hybrid hydrogels). Table 2 presents the kinetic models used in the analysis.

This comprehensive analysis using multiple kinetic models provided insights into the mechanisms governing vitamin C release in each of the tested systems.

## 3. Results and Discussion

### 3.1. Physicochemical Characterization

**Figure 2:**
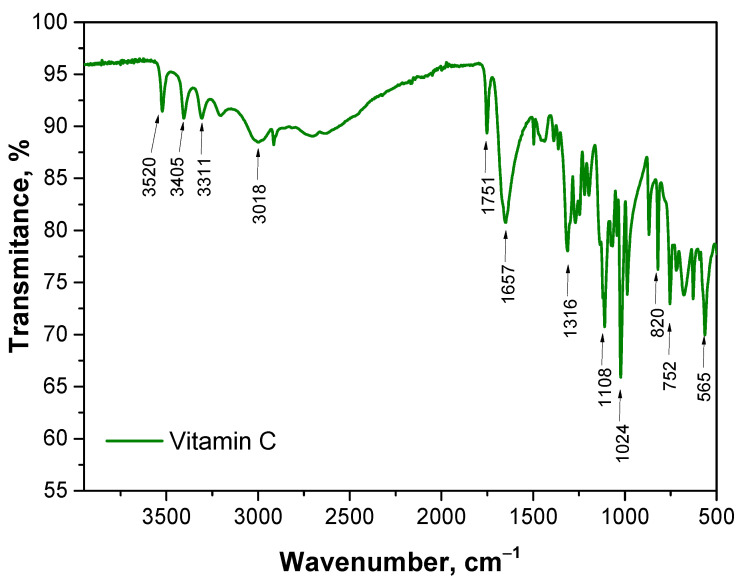
The FT IR spectrum of vitamin C.

**Figure 3:**
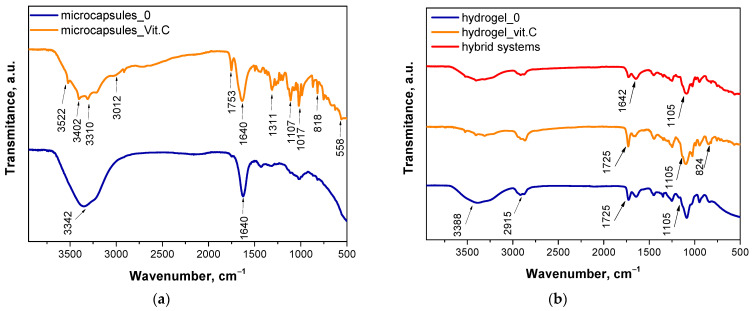
FT-IR spectroscopy spectra of alginate microcapsules (**a**); hydrogel systems (**b**).

The aim of this study was to analyze the chemical structure of various samples using FT-IR spectroscopy to identify key functional groups and assess differences due to the presence of vitamin C. The FT-IR spectrum of vitamin C is presented in Figure 2. The results obtained are consistent with the results of other researchers [26,27]. The spectra of polymeric materials are presented in Figure 3.

For the Microcapsules_0 sample, which consists of sodium alginate-based microcapsules without vitamin C, the FT-IR spectrum showed characteristic peaks for alginate. A broad band at wavenumber 3342 cm^−1^ was observed, which can be attributed to the stretching vibrations of hydroxyl (O-H) groups. This is typical for polysaccharides such as alginate, which contains numerous hydroxyl groups responsible for hydrogen bonding. The intense absorption band at 1640 cm^−1^ corresponds to the asymmetric stretching of carboxylate groups (COO^−^), indicating the presence of alginate salts. Additionally, a peak at 1428 cm^−1^ is assigned to the symmetric stretching of COO^−^, which is also characteristic of alginate. The absence of other significant peaks confirms that the sample does not contain any additional substances, such as vitamin C. In the case of the Microcapsules_Vit.C sample, which contains microcapsules with vitamin C, the FT-IR spectrum shows not only the characteristic peaks for alginate but also additional bands indicating the presence of vitamin C. Notably, a distinct peak appears at 1753 cm^−1^, which can be attributed to the stretching of the carbonyl group (C=O), characteristic of ascorbic acid. The appearance of this band confirms the presence of vitamin C in the alginate matrix. The presence of vitamin C also affects the bands at wavenumbers 3522 cm^−1^, 3405 cm^−1^, 3310 cm^−1^ and 3012 cm^−1^, indicating potential interactions between the hydroxyl groups of vitamin C and alginate, which may suggest hydrogen bonding between these components.

The FT-IR spectra for the hydrogel samples also reveal differences depending on the presence of vitamin C. For the Hydrogel_0 sample, which is a PVA-based hydrogel without vitamin C, the characteristic bands of PVA are clearly visible. The main peak at wavenumber 3388 cm^−1^ corresponds to the stretching of hydroxyl (O-H) groups, which is typical for PVA, a polymer rich in hydroxyl groups responsible for forming hydrogen bonds. A peak at 1448 cm^−1^ corresponds to the stretching of C-H bonds, characteristic of polyvinyl alcohol. The absence of additional peaks in the spectrum confirms that this sample does not contain active substances like vitamin C. In the Hydrogel_Vit.C sample, where the hydrogel contains vitamin C, additional peaks are observed compared to the hydrogel without vitamin C. A peak at 1725 cm^−1^, attributed to the carbonyl group (C=O) of ascorbic acid, is a key indicator of the presence of vitamin C in the sample. Similar to the microcapsules, the band at 3388 cm^−1^ is altered, suggesting interactions between vitamin C and PVA, likely in the form of hydrogen bonds. The final sample, the hybrid system, which is a hydrogel containing microcapsules with vitamin C, exhibits bands characteristic of both the hydrogel and the microcapsules containing vitamin C. The spectrum shows a peak at 1725 cm^−1^, confirming the presence of vitamin C in the hybrid system, although the intensity of this band is lower compared to the Hydrogel_Vit.C sample. This suggests that vitamin C is encapsulated within the microcapsules, limiting its direct interaction with the hydrogel matrix. Additionally, the spectrum shows bands associated with both alginate and PVA, confirming the complex structure of this system.

**Figure 4:**
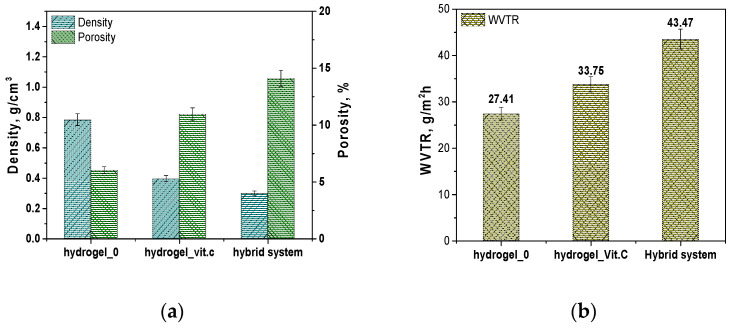
Results of hydrogel characteristics: porosity and density (**a**); water vapor transmission rate (**b**).

Next, results are presented to determine the porosity density and vapor permeability of the hydrogel systems and the hybrid carrier. The results are presented in Figure 4.

The results regarding density and porosity show significant differences between the three systems analyzed. The base hydrogel (Hydrogel_0) exhibits the highest density, measuring 0.78356 g/cm^3^, and the lowest porosity, at 6.015%. The introduction of vitamin C into the hydrogel leads to a significant decrease in density—Hydrogel_Vit.C has a density of 0.3955 g/cm^3^, indicating a reduction in the compactness of the hydrogel structure. The porosity of this system nearly doubles compared to the base hydrogel, reaching 10.946%. The lowest density was observed in the hybrid system, which contains microcapsules with vitamin C, with a value of 0.29851 g/cm^3^, indicating the most open structure. The porosity of this system is the highest, at 14.094%. These results suggest that the addition of vitamin C, whether in dissolved form in the hydrogel or encapsulated in microcapsules, leads to significant structural changes in the hydrogel. The increase in porosity may be due to the presence of a greater amount of internal spaces in the material, which may facilitate better release of active substances. Vitamin C, as an acidic substance, can affect the crosslinking of the polymer by interacting with functional groups in the hydrogel matrix, which can lead to the formation of additional spaces in the structure of the material. Increased porosity may result from such interactions, which promote the formation of internal pores and channels. The presence of vitamin C can also affect the flexibility and organization of the hydrogel network, resulting in changes in its internal structure. This kind of structure promotes easier release of active substances, as more free spaces allow for better transport and diffusion of molecules. Similar structural relationships, related to the presence of vitamin C, were observed in a study by Guo et al. In their work, chitosan hydrogels containing vitamin C (CSVC) were characterized by pores of larger size compared to pure chitosan hydrogel. The authors suggest that vitamin C affected the dispersion of chitosan throughout the hydrogel system, which promoted the formation of larger pores. In addition, CSVC hydrogels lost moisture more easily during freeze-drying, leading to an expansion of the branching structure and an increase in pore size. These observations are consistent with our results, indicating that the addition of vitamin C can affect the hydrogel structure by increasing its porosity and available internal spaces [28].

The results of the water vapor transmission rate (WVTR) tests indicate that the base hydrogel (Hydrogel_0) has the lowest WVTR value, measuring 27.41 g/m^2^/h. After the addition of vitamin C (Hydrogel_Vit.C), the WVTR increases to 33.75 g/m^2^/h, suggesting that the presence of vitamin C enhances the permeability of the hydrogel. The highest permeability was observed in the hybrid system, with a WVTR value of 43.47 g/m^2^/h. This high water vapor permeability in the hybrid system may result from the presence of microcapsules, which further contribute to the structural openness and porosity of the system, promoting more efficient moisture exchange. As mentioned above, this result is due to the effect of vitamin C on the structure of the hydrogel. The addition of vitamin C increases the porosity of the hydrogel, creating more internal spaces in its matrix. The additional pores and channels allow water vapor to penetrate more easily, resulting in higher permeability. In other words, the structural modifications associated with the presence of vitamin C increase the hydrogel’s ability to transmit water vapor by expanding the available pathways for its movement. The notable decrease in density and increase in porosity in both the sample with vitamin C and the hybrid system suggest that these materials may interact more effectively with their environment, especially in terms of the controlled release of active substances. The increased water vapor transmission rate (WVTR) also indicates potentially improved functional properties in these materials, particularly in medical applications such as dressings, where proper moisture exchange with the surrounding environment is required.

The relationship between density, porosity, and water vapor permeability (WVTR) is widely recognized as a key factor for materials such as hydrogels and microcapsules, which have applications in controlled release and medical applications such as dressings. The observed decrease in the density of hydrogel samples with a concomitant increase in porosity and an increase in WVTR values is due to the fact that materials with lower density have more empty spaces (pores), allowing water vapor and active substances to flow more easily. Structural changes in cellulose-based polymer films were demonstrated by Niu et al. The different composition affecting the hydrophilic nature of the materials was associated with a change in physicochemical properties. Again, a close relationship of a decrease in density with an increase in porosity was demonstrated. In addition, these relationships were also associated with water absorption and water vapor permeability, similarly to the results presented above [29]. Hoch et al., in turn, proved that permeability is related to the degree of hydration of the material under study and its internal structure [30]. Interestingly, internal structure parameters related to material density and permeability have also been studied in the packaging industry. Chalykh et al. proposed a model showing the relationship of permeability with water vapor diffusion coefficients in the pore volume [31]. In turn, Chavda et al. proved that fluid sorption and permeability are closely related to the degree of crosslinking of the polymer material. These results can be applied to the obtained hydrogel materials with reduced density. As shown, the lower crosslinking density of polymer chains influences their looser arrangement, which is related to properties such as sorption and porosity. Thus, the obtained results are consistent with previously presented scientific reports [32].

**Figure 5:**
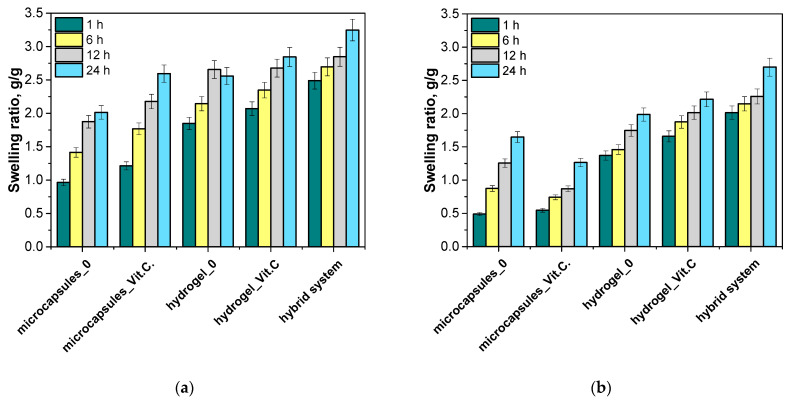
Results of analysis of the sorption capacity of the obtained carriers in distilled water (**a**); in SBF liquid (**b**).

Next, the sorption capacity was analyzed in two different environments: distilled water and SBF fluid to reflect different biological conditions. The aim was to determine the degree of swelling of the tested samples at different incubation times (1 h, 6 h, 12 h, 24 h), depending on the type of media. The results of the analysis are presented in Figure 5.

Sample microcapsules without vitamin C exhibited the lowest degree of swelling at all time points, which is consistent with expectations, as they do not contain vitamin C or other active substances that could affect fluid absorption. Their swelling degree in distilled water and SBF ranged from approximately 0.5 to 1.0 g/g after 24 h of incubation. Next, microcapsules_Vit.C showed a significantly higher degree of swelling compared to the microcapsules without vitamin C, which may be due to interactions between vitamin C and water, causing the alginate structure to expand. The degree of swelling of the microcapsules containing vitamin C reached approximately 1.5–2.0 g/g in both fluids after 24 h. In turn, hydrogel without vitamin C demonstrated a medium level of swelling, with values around 1.0–1.5 g/g after 24 h. Compared to the microcapsules, the hydrogel exhibited a greater ability to absorb fluids, which can be attributed to the hydrophilic properties of poly(vinyl alcohol) (PVA), which forms the hydrogel. Hydrogel modified with vitamin C exhibited a much higher degree of swelling than Hydrogel_0. Vitamin C, due to its hydrophilic nature, can further increase the material’s fluid absorption capacity, as reflected in the results, where the degree of swelling reached approximately 2.5–3.0 g/g in both fluids after 24 h. Interestingly, differences in the degree of swelling between Hydrogel_0 and the Hydrogel_Vit.C were not constant throughout the study period. At some time points, both samples showed similar absorption, which may suggest that structural changes caused by the presence of vitamin C, such as increased porosity, may act differently depending on the degree of water saturation of the hydrogel. The swelling values for Hydrogel_Vit.C eventually reached about 2.5–3.0 g/g after 24 h, with similar results for both samples at 12 h of testing, indicating the influence of the degree of saturation. This effect was observed only for the sample placed in distilled water. The hybrid system showed the highest degree of swelling among all tested samples, reaching values of around 3.0–3.5 g/g after 24 h. The high sorption capacity of this system results from the combined absorption properties of the hydrogel and the vitamin C-containing microcapsules.

The sorption capacity tests demonstrate that the introduction of vitamin C, both in dissolved form in the hydrogel and encapsulated in microcapsules, significantly increases the carriers’ ability to absorb fluids. The hybrid system showed the highest sorption capacity, making it potentially the most effective system for applications requiring intense absorption, such as wound dressings or controlled release systems for active substances.

The results of the hydrogels’ sorption capacity clearly correlate with the previous analyses of their density and porosity. The base hydrogel (Hydrogel_0), characterized by the highest density and lowest porosity, demonstrated a limited ability to swell, which can be attributed to its fewer pores available for fluid absorption. The introduction of vitamin C (Hydrogel_Vit.C) significantly reduced the density and simultaneously increased the porosity, which directly translated into an increased fluid absorption capacity—Hydrogel_Vit.C exhibited a much higher degree of swelling. The highest sorption capacities were observed in the hybrid system, which indicates a further increase in porosity and reduction in density, promoting even greater fluid absorption. Thus, the results suggest that lower density and higher porosity of hydrogels directly enhance their sorption capacity.

Moreover, the sorption coefficients for all tested samples were higher in distilled water compared to simulated body fluid (SBF). This is because distilled water does not contain ions or compounds that could interact with the hydrogel matrix. In contrast, SBF contains various ions, such as Na^+^, Cl^−^, and Ca^2+^, which can interact with the polymer network of the hydrogels and influence their fluid absorption capacity. These interactions may stabilize the hydrogel structure, limiting its ability to swell and absorb fluids compared to distilled water, where such restrictions are not present.

### 3.2. Incubation Analysis in Simulated Body Fluids

**Figure 6:**
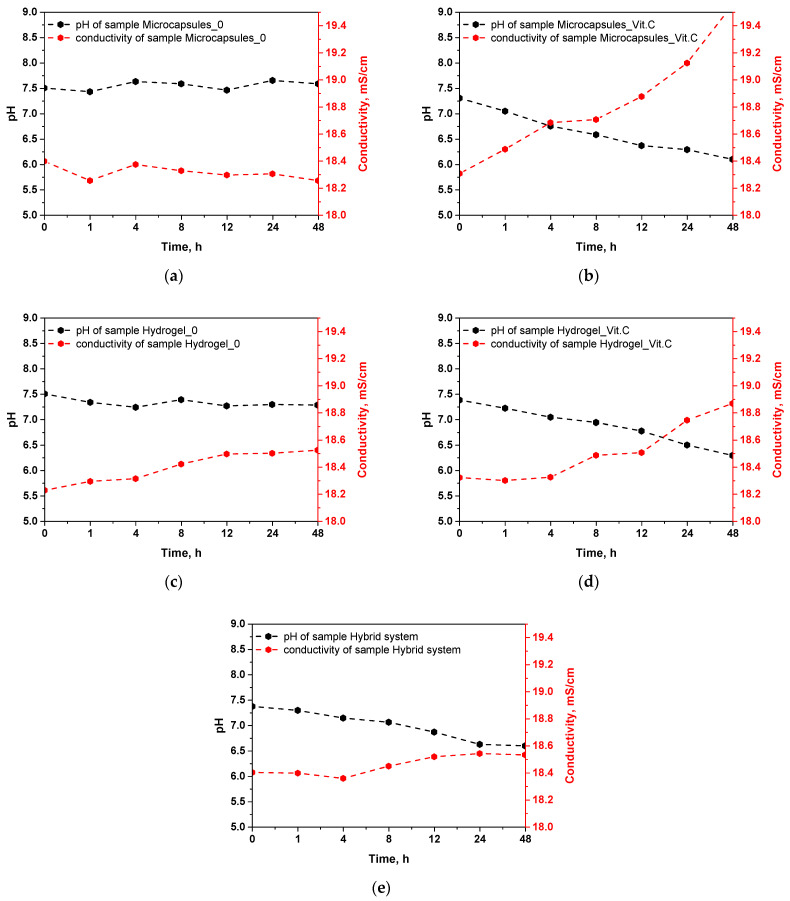
Changes in conductivity and pH during incubation testing of samples: microcapsules_0 (**a**); microcapsules_Vit.C (**b**); hydrogel_0 (**c**); hydrogel_Vit.C (**d**), hybrid system (**e**).

The results of the incubation of microcapsules and hydrogels in simulated body fluid (SBF) allow for an evaluation of the stability of these materials and their ability to release active substances, particularly vitamin C. The measured parameters, such as pH and ionic conductivity, reflect the changes occurring in the samples during incubation, providing insight into the behavior of these systems in conditions simulating a biological environment. The results of this analysis are presented in Figure 6.

The pH stability during incubation for the samples of microcapsules without vitamin C and the base hydrogel indicates their relative chemical stability. Both the microcapsules and the hydrogel without vitamin C show no significant changes in pH, suggesting no notable interactions with the SBF fluid or the release of any soluble substances. This behavior is consistent with previous results regarding density and porosity, where it was found that these samples exhibit higher density and lower porosity. Therefore, it can be assumed that in these materials, the higher-density structure limits their interaction with the surrounding environment, thus reducing their ability to release ions or active substances. In the case of the samples containing vitamin C, a marked decrease in pH during incubation is observed. This is particularly evident in the microcapsules with vitamin C, where the pH drops to 6.097 after 48 h. A similar trend is observed in the vitamin C hydrogel and the hybrid system, where the pH drops to 6.293 and 6.597, respectively. The decrease in pH can be attributed to the gradual release of vitamin C, which is ascorbic acid, and upon dissolving in the medium, it leads to acidification of the environment. This behavior aligns with earlier results on sorption capacity, which showed that vitamin C-containing systems have an increased ability to absorb fluids, which may promote faster release of vitamin C.

Ionic conductivity reflects the release of ions from the tested materials or their degradation in the SBF environment. Samples of microcapsules without vitamin C and the base hydrogel exhibit stable conductivity in the range of 18.2–18.4 mS/cm, again suggesting their high chemical stability and lack of significant structural changes during incubation. Low conductivity is consistent with earlier results, indicating their low porosity and higher density, which limits interactions with the surrounding environment and prevents ion release. In the samples containing vitamin C, an increase in conductivity is observed over time, particularly for the microcapsules with vitamin C, where the conductivity rises to 19.553 mS/cm after 48 h. Increased conductivity indicates the release of ions from the microcapsules, which may be related to the release of vitamin C and the partial degradation of the capsule material. A similar increase in conductivity is noted for the vitamin C hydrogel (18.868 mS/cm) and the hybrid system (18.532 mS/cm), further confirming that vitamin C and other ionic substances are gradually being released from these systems. The increased conductivity in vitamin C-containing samples correlates with the results on porosity. Higher porosity, especially in the hybrid system, promotes greater ion exchange between the samples and the incubation fluid, leading to increased conductivity. In addition, the change in conductivity values in the tested incubation fluids confirms that the polymer systems were able to effectively release vitamin C and ion exchange with the environment, which confirms their potential in controlled release of the active substance.

The results of the incubation in simulated body fluid (SBF) clearly indicate differences in the behavior of the tested samples depending on their composition. Samples containing vitamin C show more significant changes in both pH and ionic conductivity, suggesting their ability to gradually release vitamin C and possibly other ionic compounds. On the other hand, samples without vitamin C placed in the incurable fluid do not change its pH and conductivity. Therefore, it can be concluded that the changes in these parameters are mainly related to the presence of vitamin C.

### 3.3. Microscopic Observations and Surface Roughness

**Figure 7:**
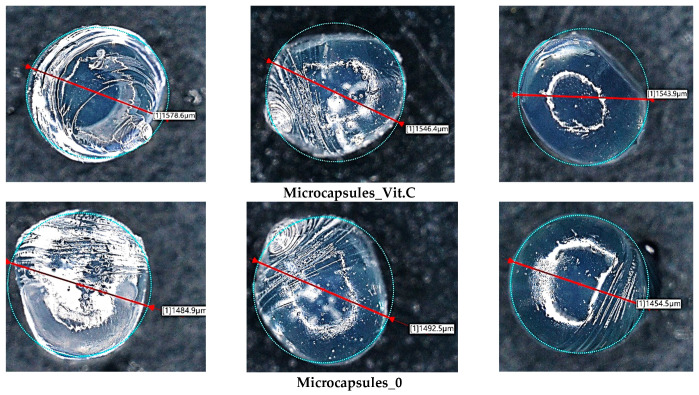
Images of alginate microcapsules without and with vitamin C (magnification ×20).

**Figure 8:**
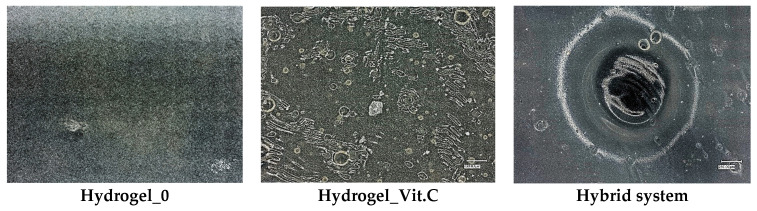
Images of hydrogels without and with vitamin C (magnification ×100).

Subsequently, microscopic observations were made of the resulting systems. In the case of capsules, their average size was determined. The results are presented in Figure 7. Then, the surface morphology of the hydrogel materials was visualized (Figure 8).

The irregular rod-like and sphere-like structures seen in Figure 6 are characteristic of the microcapsules formed when alginate is infused into calcium chloride solution. These irregularities are due to the rapid gelation process that occurs when a drop of alginate detaches from the tip of the needle. As each droplet detaches from the needle, its surface begins to crosslink immediately upon contact with the calcium ions in the solution, resulting in local rippling or minor deformations, particularly at the point of droplet detachment. Rapid crosslinking of the droplet surface can lead to the formation of small protuberances or ripples, which result in the observed irregularities in the microcapsule structure.

The basic hydrogel has a relatively smooth and uniform surface, suggesting the absence of defects created during photopolymerization. In contrast, the vitamin C-containing hydrogel appears rougher and more irregular, which may be related to interactions of vitamin C with the polymer matrix, leading to changes in the surface structure. The most pronounced changes are observed in the case of the hybrid system, which contains microcapsules with vitamin C. The photo clearly shows a more complex and rougher surface, indicating the presence of microcapsules inside the hydrogel structure. No major changes in the shape of the microcapsules were observed. They are mostly close to spherical in shape. The irregularities are due to the crosslinking process. Vitamin C-containing capsules are characterized by a larger diameter, with little change.

**Figure 9:**
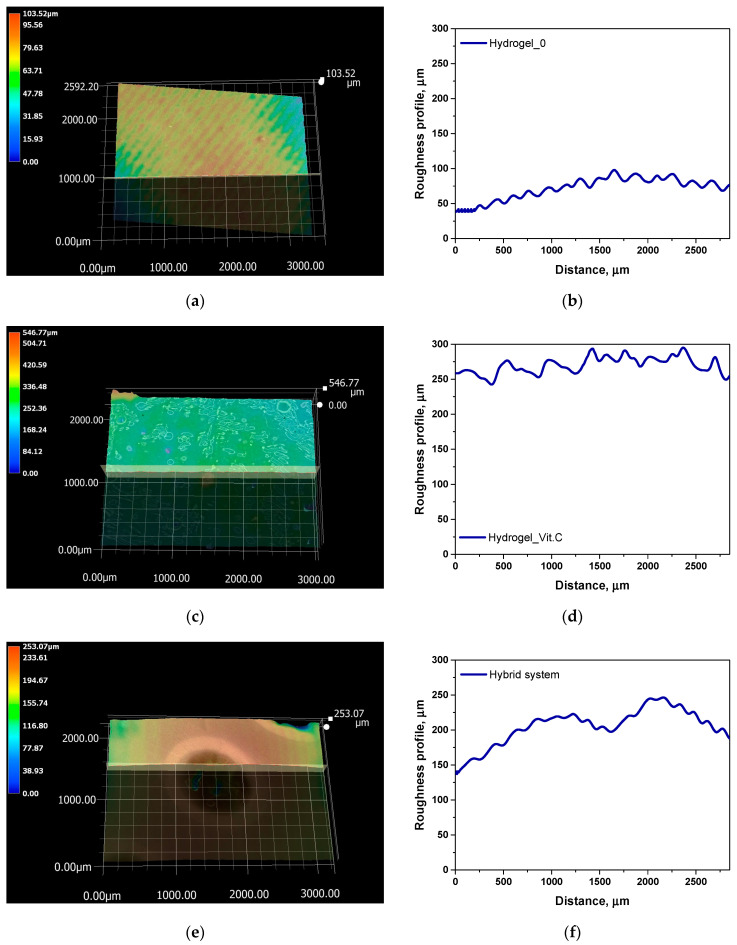
Results of surface roughness analysis for hydrogel_0 samples (**a**,**b**); hydrogel_Vit.C (**c**,**d**); hybrid system (**e**,f).

Next, a roughness profile analysis was conducted for the hydrogel samples. The results are presented in Figure 9 and Table 3.

The analysis of surface roughness measurements, combined with previous data on porosity and density, allows for conclusions regarding the relationship between surface structure and the functional properties of the studied systems. The average roughness (Ra) and maximum profile height (Rz) values revealed significant differences between the samples, reflecting the impact of the presence of vitamin C and microcapsules on the surface topography. Relatively low Ra and Rz values in the base hydrogel suggest that its surface is more smooth, likely due to the lower porosity of this system. The base hydrogel also exhibited the highest density and lowest porosity, which limited its ability to absorb fluids, as reflected in the lower roughness values. Reduced roughness and lower porosity lead to a smaller surface area available for interaction with the environment, which may be disadvantageous in applications requiring intensive fluid exchange, such as wound dressings. The addition of vitamin C in the hydrogel significantly increased both the surface roughness (Ra = 14.16 µm, Rz = 98.12 µm) and porosity, likely due to interactions between vitamin C and the hydrogel’s polymer network. The increased surface roughness is consistent with the porosity results, indicating a more open and branched structure in this hydrogel, which facilitates greater fluid absorption capacity. The higher porosity and surface roughness of the vitamin C-containing hydrogel may promote better interaction with the biological environment, which is critical for applications such as drug delivery systems or medical wound dressings. The highest Ra (20.15 µm) and Rz (89.51 µm) values were recorded for the hybrid system, which aligns with the presence of microcapsules within the hydrogel structure. The inclusion of microcapsules resulted in a significant increase in the porosity of this system, as confirmed by previous density and porosity measurements. The increased roughness in the hybrid system suggests that the surface of the microcapsule-containing hydrogel is much more uneven, which could provide a larger contact area with fluids and enable better control of active substance release. The high porosity and roughness of this system not only improve sorption capacity but may also influence the efficiency of active substance release, making this system promising for applications requiring controlled release, such as drug delivery or long-term wound dressings. In summary, the analysis indicates a strong correlation between surface roughness and the porosity of the studied systems. The increase in roughness, resulting from the modification of hydrogels with vitamin C and microcapsules, enhances sorption properties and potentially improves bioadhesion, which is crucial for medical applications.

**Figure 10:**
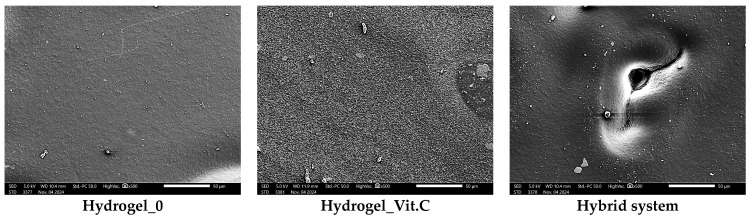
Results of observations from scanning electron microscope.

Figure 10 shows images obtained from SEM observations, which are consistent with digital microscope analysis and roughness measurements. The basic hydrogel shows a relatively smooth and uniform surface, indicating a structure without additional modifications. In contrast, the vitamin C-containing hydrogel shows increased texture irregularity, likely due to interactions between vitamin C and the polymer matrix, which alter the surface structure. At the same time, an increase in the corrugation of the entire surface can be seen. No defects in the polymer matrix were observed. The most noticeable changes are observed in the hybrid system containing microcapsules with vitamin C. SEM images reveal the presence of pores and a more irregular structure. These results are consistent with the observations presented above regarding surface roughness.

### 3.4. Evaluation of Release Kinetics Under Static and Dynamic Conditions

Subsequently, a study on the release of vitamin C from the microcapsules and hydrogels was conducted. To measure the release, a reaction with ferric chloride and phenanthroline was used. 

**Figure 11:**
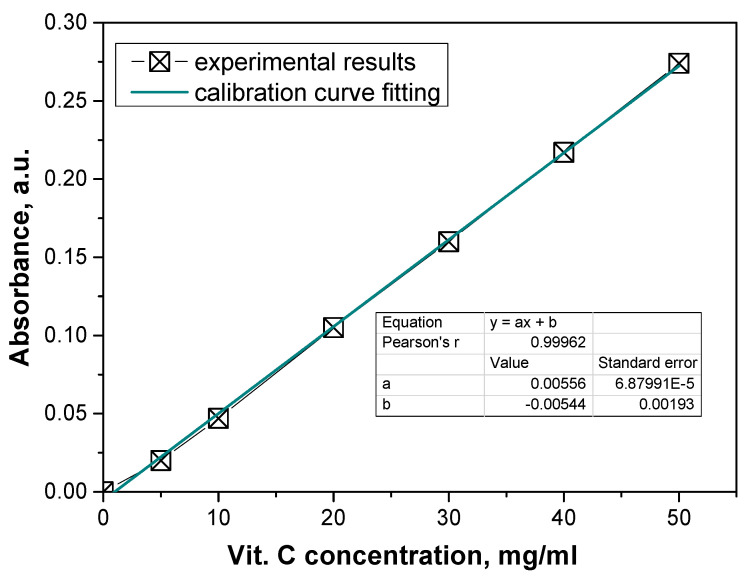
Calibration curve of the determined vitamin C.

This study began by determining a calibration curve to quantify the release of vitamin C. The calibration curve was constructed by measuring the absorbance of solutions with known concentrations of vitamin C. The concentration of vitamin C (in mg/L) was plotted on the *x*-axis, while the corresponding absorbance values (measured at the wavelength specific to the Fe^2+^-phenanthroline complex) were plotted on the *y*-axis (Figure 11).

**Figure 12:**
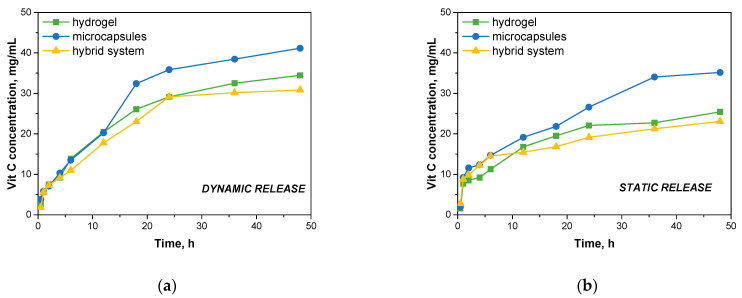
Results of vitamin C release through dynamic (**a**) and static processes (**b**).

This calibration curve provides a linear relationship between the concentration of vitamin C and the absorbance, which allows for the determination of unknown concentrations of vitamin C in the release study based on their absorbance values. Figure 12 then presents the results of vitamin C release at specific time intervals in a dynamic and static process.

Under dynamic conditions, it was observed that the microcapsules showed the highest rate of vitamin C release. After 48 h, the microcapsules released 41.137 mg/mL of vitamin C, indicating rapid diffusion of the active ingredient from the microcapsule matrix. The rapid release can be explained by the relatively small structure of the microcapsules, which are subjected to continuous mechanical forces under dynamic conditions, promoting the swelling of the capsules and facilitating the release of vitamin C into the medium. Hydrogels showed a slightly slower release rate compared to microcapsules. After 48 h, the hydrogels released 34.448 mg/mL of vitamin C. The slower release from the hydrogels may be related to the more crosslinked structure of the hydrogel matrix, which controls the diffusion of vitamin C. However, the dynamic experimental conditions favored greater contact between the hydrogel surface and the incubation fluid, which improved the diffusion of the active ingredient, although it was still slower than with microcapsules. The lowest release values were recorded for the hybrid system, which released 30.836 mg/mL of vitamin C after 48 h. The hybrid system, consisting of microcapsules embedded in a hydrogel, showed the most controlled release of vitamin C. The hydrogel structure appears to provide an additional barrier that limits the rate of vitamin C release from the microcapsules. This composite structure provides a more gradual release of the active ingredient, which may be beneficial in applications that require prolonged action, such as dressings or drug delivery systems.

Under static conditions, where no mechanical mixing was used, the rate of vitamin C release was noticeably slower in all systems tested. After 48 h, the microcapsules released 35.177 mg/mL of vitamin C, which was still the highest value compared to the other systems. The lack of mechanical agitation slowed the diffusion process, but the microcapsules were able to release the active ingredient relatively quickly due to their small structure. The hydrogels released 25.437 mg/mL of vitamin C after 48 h, a lower value compared to the dynamic process. Under static conditions, where contact with the incubation fluid was more limited, the hydrogels showed slower release of the active ingredient, which may be explained by the higher density of the hydrogel matrix, which limited the migration of vitamin C into the environment. The hybrid system also showed the lowest release rate in the static process, with a result of 23.085 mg/mL of vitamin C after 48 h. Such a result confirms that the complex structure of the hybrid system, in which the hydrogel further surrounds the microcapsules, significantly delays the release of vitamin C. Under static conditions, where there are no additional mechanical forces to promote diffusion, the hydrogel barrier works even more effectively, leading to a slower and more controlled release.

The results show significant application potential in extended-release active ingredient delivery systems. Compared to other developed systems, a relatively prolonged release profile of vitamin C was achieved. Systems described in the literature are characterized by the undesirable effect of sudden ejection of the substance, which may limit their application where controlled, prolonged release of the active substance is required. In a study conducted by Calzado-Delgado on poly(vinyl alcohol) (PVA) nanofiber media, a rapid release of vitamin C was observed—as much as 63% of the total vitamin content was released within the first hour [33]. Similarly, in the work of Shing et al., an orally dissolvable film (ODF) containing vitamin C and catechin was studied. The results of this study also indicate an intense rapid release effect, as more than 70% of vitamin C was released in simulated body fluid within the first 30 min. Such a rapid release profile makes this system more suitable for rapid delivery of antioxidants, but it does not meet the objectives for prolonged release [34]. Also, in a study by Abdullach et al. on hydrogel systems containing avocado seed protein, there was a sudden ejection of vitamin C during the initial phases of release. Although the authors describe the use of this system as a potential carrier for bioactive compounds, the resulting release profile indicates the difficulty of achieving prolonged action without changing the composition and structure of the hydrogel [24].

**Figure 13:**
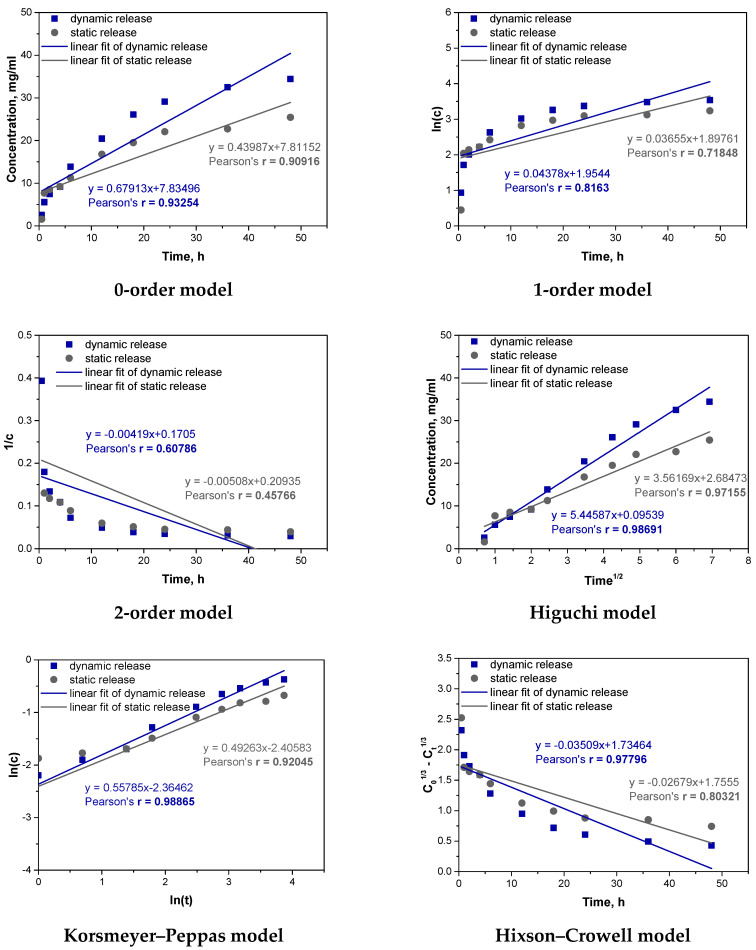
Fitting experimental results to different kinetic models for hydrogel_Vit.C sample.

In order to better understand the process of vitamin C release from the obtained carriers, fitting of experimental results to different kinetic models was also carried out. The results of fitting the results for the Hydrogel_Vit.C sample are shown below in Figure 13 and Table 4.

The analysis of the kinetics of vitamin C release from the hydrogel containing vitamin C was performed using six kinetic models. The best fit to the experimental results was obtained for the Higuchi and Korsmeyer–Peppas models. The Higuchi model, which assumes that the release of the substance is primarily controlled by diffusion through a porous matrix, showed very high correlation coefficients for both dynamic (r = 0.98691) and static (r = 0.97155) conditions. This result suggests that diffusion is the dominant mechanism controlling the release of vitamin C from the hydrogel in both cases. Under dynamic conditions, where mechanical agitation is applied, the diffusion process is enhanced by the movement of the fluid, facilitating faster penetration of vitamin C through the hydrogel matrix. In contrast, in static conditions, diffusion occurs more slowly, as reflected by the slightly lower correlation coefficient, though diffusion still plays a key role in controlling the release process. The second model that well described the kinetics of vitamin C release from the hydrogel was the Korsmeyer–Peppas model. This model is useful for systems where the release mechanism is more complex, involving both diffusion and polymer matrix swelling. The correlation coefficient for dynamic release was r = 0.98865, and for static release, it was r = 0.92045, indicating that this model also fits the data very well. An important parameter in the Korsmeyer–Peppas model is the exponent “n”, which provides information about the mechanism controlling the release. When “n” is around 0.5, the process is dominated by Fickian diffusion (a simple diffusion mechanism). When “n” exceeds 0.5, it suggests that the release is controlled by both diffusion and swelling of the matrix, indicating a more complex mechanism. In the case of the vitamin C hydrogel, the value of “n” obtained from the Korsmeyer–Peppas model was greater than 0.5, suggesting that, in addition to diffusion, matrix swelling also plays a significant role in controlling the release. Mechanical agitation in the dynamic process likely enhanced this effect, which explains the better fit of the model under these conditions.

Both the Higuchi and Korsmeyer–Peppas models confirm that diffusion is the key mechanism controlling the release of vitamin C from the hydrogel, regardless of the conditions. However, differences in model fit between dynamic and static release indicate that these processes occur at different rates depending on the conditions. In dynamic conditions, where mechanical agitation facilitates swelling and increases contact between the hydrogel matrix and the medium, the release mechanism becomes more complex, involving both diffusion and swelling, which explains the better fit of the Korsmeyer–Peppas model. In static conditions, without mechanical assistance, the swelling process is slower, and diffusion is more strictly controlled by the porous structure of the hydrogel, which is better reflected by the Higuchi model.

**Figure 14:**
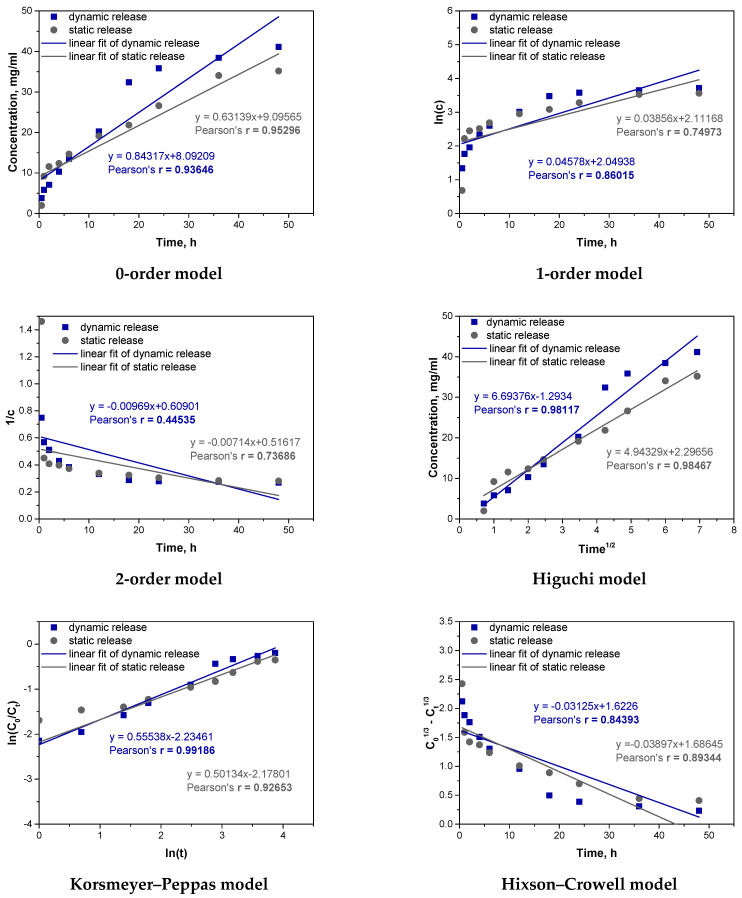
Fitting experimental results to different kinetic models for microcapsules_Vit.C. sample.

Analogous fitting was then carried out for the microcapsules_Vit.C sample. The results are presented in Figure 14 and Table 5.

For the microcapsules_Vit.C sample, the analysis of Pearson’s correlation coefficients indicates that under dynamic conditions, the Korsmeyer–Peppas model (r = 0.99186) and the Higuchi model (r = 0.98117) best describe the release of vitamin C. The high correlation for the Korsmeyer–Peppas model, with an “n” value above 0.5, suggests that both diffusion and swelling significantly influence release, as mechanical agitation enhances the swelling of the alginate matrix. The Higuchi model, though also showing high correlation, primarily reflects diffusion without accounting for swelling, explaining its slightly lower fit. In static conditions, the Higuchi model (r = 0.98467) and the zero-order model (r = 0.95296) provide the best fit. The high fit of the Higuchi model confirms that diffusion is the main mechanism without significant swelling due to the absence of mechanical agitation. The zero-order model indicates a stable release rate, suggesting a steady diffusion process, especially in the early stages. Comparing dynamic and static processes, mechanical agitation in dynamic conditions promotes swelling and a complex release mechanism, fitting well with the Korsmeyer–Peppas model. In contrast, static conditions favor diffusion as the dominant mechanism, consistent with the Higuchi model’s fit. Thus, release mechanisms vary with conditions: dynamic conditions involve both diffusion and swelling, while static conditions are dominated by diffusion alone.

**Figure 15:**
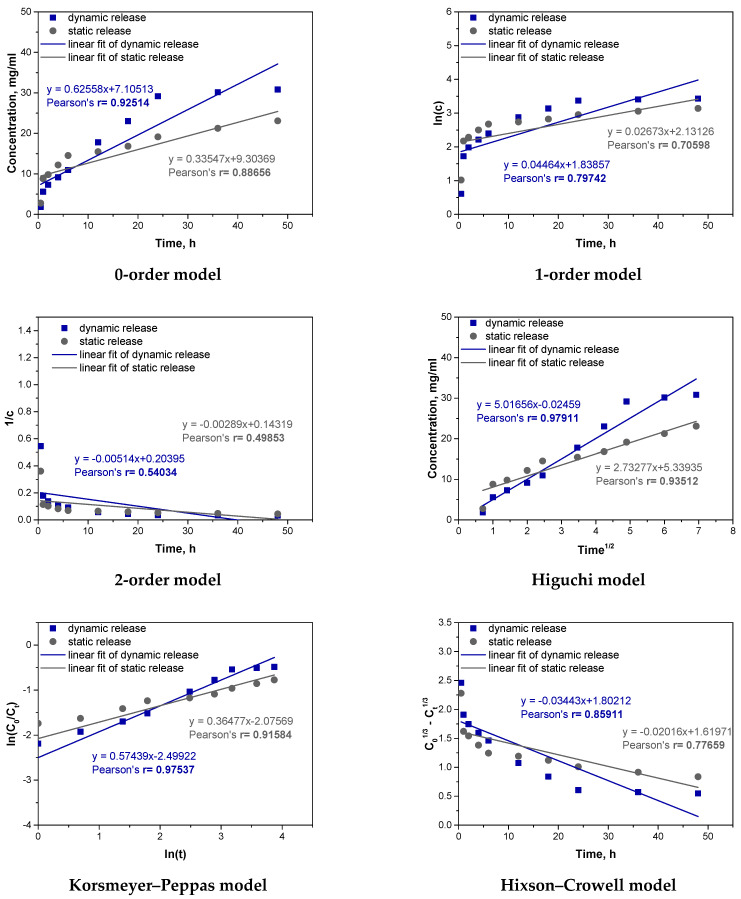
Fitting experimental results to different kinetic models for hybrid system.

Next, similar fitting was carried out for a sample of the hybrid system. The results are presented in Figure 15 and Table 6.

For the hybrid system (a hydrogel containing vitamin C microcapsules), the best kinetic fit was obtained for the Higuchi model (r = 0.97911 dynamic, r = 0.93512 static) and the Korsmeyer–Peppas model (r = 0.97537 dynamic, r = 0.91584 static). This suggests that the release mechanism is complex, involving both diffusion and swelling. In dynamic conditions, where agitation enhances the interaction with the medium, swelling plays a larger role, as reflected by the Korsmeyer–Peppas model’s better fit. In static conditions, limited swelling emphasizes diffusion, aligning well with the Higuchi model. The release rate of vitamin C in the hybrid system was slower compared to the microcapsule and hydrogel samples, with 30.836 mg released in dynamic and 23.085 mg in static conditions, versus higher releases in the other samples. This slower, more controlled release results from the dual barriers of the hydrogel and microcapsules, creating additional resistance to diffusion. Compared to previous analyses, the hybrid system’s structure offers a slower, more sustained release, unlike the microcapsules, which provided a faster, diffusion-dominated release. The hybrid system’s dual-barrier structure makes it suitable for applications requiring prolonged, stable release of active compounds, valuable in drug delivery contexts.

## 4. Conclusions

This study focused on the synthesis and characterization of polymeric materials as vitamin C carriers. Basic hydrogels were synthesized using a photopolymerization process. Polyethylene glycol diacrylate (PEGDA) was used as a crosslinking agent, while 2-hydroxy-2-methylpropiophenone was used as a photoinitiator. Polyvinyl alcohol (PVA) was chosen as the main polymer to form the hydrogel matrix. The hydrogels without additives and containing vitamin C were tested. In addition, a hybrid system was tested that contained vitamin C encapsulated in capsules made from sodium alginate.

The analysis of density and porosity revealed that systems with lower density and higher porosity, such as the hybrid system, exhibited better sorption capacities and more controlled vitamin C release. Lower density and higher porosity promoted increased fluid absorption, which affected the release rate of the active substance. The water vapor transmission rate (WVTR) results showed that modifying the hydrogel with vitamin C and introducing microcapsules increased permeability, which may be beneficial for applications such as medical dressings. The highest permeability was observed in the hybrid system, which also had the highest porosity. These results suggest that such structures can better interact with the environment, ensuring proper moisture exchange. Sorption capacity tests confirmed that systems with higher porosity, such as the hybrid system and the vitamin C hydrogel, had greater fluid absorption capacities, which contributed to more controlled vitamin C release. The hybrid system showed the highest sorption capacity, which was mainly influenced by its low density and high porosity, which directly affected its ability to absorb substances. Incubation in SBF fluid demonstrated that systems containing vitamin C, particularly microcapsules and the hybrid system, showed significant changes in pH and conductivity, indicating that these systems influence the chemical properties of the surrounding environment. These observations confirm that vitamin C released from polymeric systems can influence changes in the pH and conductivity of the incubation fluids.

A key part of this study was the analysis of vitamin C release kinetics. For all systems, the best fit was obtained with the Higuchi model, which describes diffusion processes, and the Korsmeyer–Peppas model, which also accounts for matrix swelling. Microcapsules exhibited the fastest vitamin C release, especially in dynamic conditions, where the swelling of the alginate matrix facilitated faster diffusion. Vitamin C hydrogel showed slower release than from alginate capsules, with hydrogel swelling also having an important role under dynamic conditions, as well described by the Korsmeyer–Peppas model. In the hybrid system, which combined microcapsules with the hydrogel, the release process was the most controlled and prolonged, due to the additional barriers created by both the hydrogel and microcapsules.

In conclusion, this study showed that the structure of the carrier has a key impact on the mechanisms of vitamin C release. Microcapsules provided rapid release, while the hydrogel and hybrid system allowed for more controlled but slower release. The hybrid system, combining the advantages of both carriers, exhibited the most controlled and prolonged release, which may be particularly significant in applications such as drug delivery systems, where long-lasting and stable release of active substances is required. Moreover, in the context of the study results and the known properties of vitamin C, its role as an excipient in drug carriers can be considered, especially for medical applications requiring high sorption capacity and bioadhesion. Vitamin C, due to its hydrating properties and ability to modify the structure of the hydrogel matrix, contributes to increased porosity and permeability, which can support the release of active substances. It is possible that similar effects could be achieved by using other active substances with hydrophilic or ionic characteristics, which may interact with the polymer matrix and influence its physicochemical properties. The broad application of such substances could be beneficial in carriers for various drugs, particularly in controlled release systems, wound dressings, and implants, where high bioadhesion and enhanced absorption capacity are essential.

## Figures and Tables

**Table 1:** List of tested materials.

Sample Name	Sample Description
Microcapsules_0	microcapsules without additives
Microcapsules_Vit.C	microcapsules containing vitamin C
Hydrogel_0	hydrogel without additives
Hydrogel_Vit.C	hydrogels containing vitamin C
Hybrid system	hydrogel containing microcapsules with vitamin C

**Table 2:** The selected kinetic models to which the experimental results were fitted.

Kinetic Model	Model Equation	Description
Zero-order model	$C_{t}=C_{0}-k_{0}t$	Constant release rate, independent of concentration.
First-order model	$lnC_{t}=lnC_{0}-k_{1}t$	Release depends on the remaining concentration of the substance.
Second-order model	$\frac{1}{C_{t}}=\frac{1}{C_{0}}+k_{2}t$	The release rate changes proportionally to the square of the concentration.
Higuchi model	$Q=k_{H}t^{1/2}$	Release controlled by diffusion through a solid matrix.
Korsmeyer–Peppas model	$Q=k_{p}t^{n}$	Describes release based on diffusion and matrix erosion, where n determines the mechanism.
Hixson–Crowell model	$\left(C_{0}^{1/3}-\;C_{t}^{1/3}\right)=k_{H}Ct$	Accounts for the changing surface area and particle size during dissolution.

**Table 3:** Roughness parameters of hydrogel materials and hybrid system.

Sample Name	Ra, µm	Rz, µm
Hydrogel_0	10.57	52.13
Hydrogel_Vit.C	14.16	98.12
Hybrid system	20.15	89.51

**Table 4:** Summary of kinetic parameters for selected best-fit models for hydrogel_vit.C sample.

Kinetic Model	Dynamic Release	Static Release
Higuchi	k_H_ = 5.44587 r = 0.98691	k_H_ = 3.56169 r = 0.97155
Korsmeyer–Peppas	k_P_ = 0.09399 r = 0.98865	k_P_ = 0.09019 r = 0.92045

k_H_—rate constant in the Higuchi model; k_P_—rate constant in the Korsmeyer–Peppas model; r—Pearson’s r correlation coefficient.

**Table 5:** Summary of kinetic parameters for selected best-fit models for microcapsules_Vit.C sample.

Kinetic Model	Dynamic Release	Static Release
0-order	k_0_ = 0.84317 r = 0.93646	k_0_ = 0.63139 r = 0.95296
Higuchi	k_H_ = 6.69376 r = 0.98117	k_H_ = 4.94329 r = 0.98467
Korsmeyer–Peppas	k_P_ = 0.1070 r = 0.99186	k_P_ = 0.11327 r = 0.92653

k_0_–rate constant in the 0-order model; k_H_—rate constant in the Higuchi model; k_P_—rate constant in the Korsmeyer–Peppas model; r—Pearson’s r correlation coefficient.

**Table 6:** Summary of kinetic parameters for selected best-fit models for hybrid system.

Kinetic Model	Dynamic Release	Static Release
Higuchi	k_H_ = 5.01656 r = 0.97911	k_H_ = 2.73277 r = 0.93512
Korsmeyer–Peppas	k_P_ = 0.08215 r = 0.97537	k_P_ = 0.12547 r = 0.91584

k_H_—rate constant in the Higuchi model; k_P_—rate constant in the Korsmeyer–Peppas model; r—Pearson’s r correlation coefficient.

## Data Availability

The data that support the findings of this study are contained within the article.

## References

[1] Siepmann J., Peppas N.A. (2001). Modeling of Drug Release from Delivery Systems Based on Hydroxypropyl Methylcellulose (HPMC). Adv. Drug Deliv. Rev..

[2] Colombo P., Bettini R., Santi P., Peppas N.A. (2000). Swellable Matrices for Controlled Drug Delivery: Gel-Layer Behaviour, Mechanisms and Optimal Performance. Pharm. Sci. Technol. Today.

[3] Bhardwaj V., Hariharan S., Balasubramanian I., Lamprecht A., Kumar N., Panchagnula R., Kumar M.N.V.R. (2005). Pharmaceutical Aspects of Polymeric Nanoparticles for Oral Drug Delivery. J. Biomed. Nanotechnol..

[4] Wojcik-Pastuszka D., Krzak J., Macikowski B., Berkowski R., Osiński B., Musiał W. (2019). Evaluation of the Release Kinetics of a Pharmacologically Active Substance from Model Intra-Articular Implants Replacing the Cruciate Ligaments of the Knee. Materials.

[5] Dash S., Murthy P.N., Nath L., Chowdhury P. (2010). Kinetic Modeling on Drug Release from Controlled Drug Delivery Systems. Acta Pol. Pharm..

[6] Lindner W.D., Lippold B.C. (1995). Drug Release from Hydrocolloid Embeddings with High or Low Susceptibility to Hydrodynamic Stress. Pharm. Res..

[7] Narasimhan B., Peppas N.A. (1997). Molecular Analysis of Drug Delivery Systems Controlled by Dissolution of the Polymer Carrier. J. Pharm. Sci..

[8] Lin C.-C., Metters A.T. (2006). Hydrogels in Controlled Release Formulations: Network Design and Mathematical Modeling. Adv. Drug Deliv. Rev..

[9] Das R., Bhattacharjee C., Inamuddin A.M., Asiri A.M. (2018). 23-Hydrogel Nanocomposite for Controlled Drug Release. Applications of Nanocomposite Materials in Drug Delivery.

[10] Lin Y.-S., Tsay R.-Y. (2020). Drug Release from a Spherical Matrix: Theoretical Analysis for a Finite Dissolution Rate Affected by Geometric Shape of Dispersed Drugs. Pharmaceutics.

[11] Merchant H., Shoaib M., Tazeen J., Ismail Yousuf R. (2006). Once-Daily Tablet Formulation and In Vitro Release Evaluation of Cefpodoxime Using Hydroxypropyl Methylcellulose: A Technical Note. AAPS PharmSciTech.

[12] Wu I.Y., Bala S., Škalko-Basnet N., di Cagno M.P. (2019). Interpreting Non-Linear Drug Diffusion Data: Utilizing Korsmeyer-Peppas Model to Study Drug Release from Liposomes. Eur. J. Pharm. Sci..

[13] Heredia N.S., Vizuete K., Flores-Calero M., Pazmiño V.K., Pilaquinga F., Kumar B., Debut A. (2022). Comparative Statistical Analysis of the Release Kinetics Models for Nanoprecipitated Drug Delivery Systems Based on Poly(Lactic-Co-Glycolic Acid). PLoS ONE.

[14] Ahmed L., Atif R., Eldeen T., Yahya I., Omara A., Eltayeb M. (2019). Study the Using of Nanoparticles as Drug Delivery System Based on Mathematical Models for Controlled Release. Int. J. Latest Technol. Eng. Manag. Appl. Sci. IJLTEMAS.

[15] Li X., Liu L., Yang P., Li P., Xin J., Su R. (2013). Synthesis of Collagen-Modified Polylactide and Its Application in Drug Delivery. J. Appl. Polym. Sci..

[16] Wagner B.A., Buettner G.R. (2023). Stability of Aqueous Solutions of Ascorbate for Basic Research and for Intravenous Administration. Adv. Redox Res..

[17] Feszterová M., Kowalska M., Mišiaková M. (2023). Stability of Vitamin C Content in Plant and Vegetable Juices under Different Storing Conditions. Appl. Sci..

[18] Yin X., Chen K., Cheng H., Chen X., Feng S., Song Y., Liang L. (2022). Chemical Stability of Ascorbic Acid Integrated into Commercial Products: A Review on Bioactivity and Delivery Technology. Antioxidants.

[19] Bacha K., Chemotti C., Monboisse J.-C., Robert A., Furlan A.L., Smeralda W., Damblon C., Estager J., Brassart-Pasco S., Mbakidi J.-P. (2022). Encapsulation of Vitamin C by Glycerol-Derived Dendrimers, Their Interaction with Biomimetic Models of *Stratum corneum* and Their Cytotoxicity. Molecules.

[20] Maurya V.K., Shakya A., McClements D.J., Srinivasan R., Bashir K., Ramesh T., Lee J., Sathiyamoorthi E. (2023). Vitamin C Fortification: Need and Recent Trends in Encapsulation Technologies. Front. Nutr..

[21] Baek J., Ramasamy M., Willis N.C., Kim D.S., Anderson W.A., Tam K.C. (2021). Encapsulation and Controlled Release of Vitamin C in Modified Cellulose Nanocrystal/Chitosan Nanocapsules. Curr. Res. Food Sci..

[22] Viscovich M., Lykkesfeldt J., Poulsen H.E. (2004). Vitamin C Pharmacokinetics of Plain and Slow Release Formulations in Smokers. Clin. Nutr..

[23] Perchyonok T., Reher V., Zhang S., Oberholzer T., Massey W., Grobler S. (2014). Chitosan:Vitamin C Containing Hydrogels as a Prototype Functional Prolonged Pain Management Restorative Material In-Vitro Studies. Open J. Stomatol..

[24] Abdullah B.A., Basyigit B., Karaaslan M. (2023). Drying Technique Providing Maximum Benefits on Hydrogelling Ability of Avocado Seed Protein: Spray Drying. Foods.

[25] Szatkowski P., Flis Z., Ptak A., Molik E. (2024). Hydrogel Dressing Biomaterial Enriched with Vitamin C: Synthesis and Characterization. Int. J. Mol. Sci..

[26] Chaudhry H., Rangra N. (2023). Development and Validation of a Stability Indicating Green Analytical Method for the Simultaneous Estimation of L-Glutathione, N-Acetyl l-Cysteine and Vitamin C in Marketed Formulation Using UV—Visible Spectroscopy. Futur. J. Pharm. Sci..

[27] Claudia C., Marinoiu A., Cernatescu C. (2015). Sorption of Vitamin C on Acid Modified Clinoptilolite. Rev. Roum. Chim..

[28] Guo Y., Qu Y., Yu J., Song L., Chen S., Qin Z., Gong J., Zhan H., Gao Y., Zhang J. (2022). A Chitosan-Vitamin C Based Injectable Hydrogel Improves Cell Survival under Oxidative Stress. Int. J. Biol. Macromol..

[29] Niu X., Liu Y., King A., Hietala S., Rojas O., Pan H. (2019). Plasticized Cellulosic Films by Partial Esterification and Welding in Low-Concentration Ionic Liquid Electrolyte. Biomacromolecules.

[30] Hoch G., Chauhan A., Radke C.J. (2003). Permeability and Diffusivity for Water Transport through Hydrogel Membranes. J. Memb. Sci..

[31] Chalykh A., Pavel Z., Tatiana C., Rubtsov A., Svetlana Z. (2020). Water Vapor Permeability through Porous Polymeric Membranes with Various Hydrophilicity as Synthetic and Natural Barriers. Polymers.

[32] Chavda H., Patel C. (2011). Effect of Crosslinker Concentration on Characteristics of Superporous Hydrogel. Int. J. Pharm. Investig..

[33] Calzado-Delgado M., Guerrero-Pérez M.O., Yeung K.L. (2023). Dissolvable Topical Formulations for Burst and Constant Delivery of Vitamin C. ACS Omega.

[34] Shin H.-J., Chang J.-H., Han J.-A. (2023). Physicochemical and In-Vitro Release Characteristics of Vitamin C-Loaded Antioxidant Orally Disintegrating Films with Different Catechin Levels. Food Biosci..

